# Supplementary material for: Solar-driven upgrading of biomass by coupled hydrogenation using in situ (photo)electrochemically generated H2
Source: Nat Commun. 2023 Sep 27;14:6017. doi: 10.1038/s41467-023-41742-4 (PMC10533862; doi:10.1038/s41467-023-41742-4)
Supplement: Supplementary file 1 — Supplementary Information [file 41467_2023_41742_MOESM1_ESM.pdf]

Supplementary information

Solar-driven upgrading of biomass-derived feedstock by coupled homogeneous hydrogenation using in situ (photo)electrochemically generated H_2

Keisuke Obata,[1,2] Michael Schwarze,[3] Tabea A. Thiel,[3,4] Xinyi Zhang,[1] Babu Radhakrishnan,[1] Ibbi Y. Ahmet,[1] Roel van de Krol,[1,3] Reinhard Schomäcker[3] and Fatwa F. Abdi[1,5*]

[1] Institute for Solar Fuels, Helmholtz-Zentrum Berlin für Materialien und Energie GmbH, Hahn-Meitner-Platz 1, 14109, Berlin, Germany

[2] Department of Chemical System Engineering, School of Engineering, The University of Tokyo, Tokyo 113-8656, Japan

[3] Technische Universität Berlin, Department of Chemistry, Straße des 17. Juni 124, 10623, Berlin, Germany

[4] Leibniz Institute for Catalysis, Albert-Einstein-Straße 29a, 18059, Rostock, Germany

[5] School of Energy and Environment, City University of Hong Kong, 83 Tat Chee Avenue, Kowloon, Hong Kong SAR, China

Correspondence and requests for materials should be addressed to Fatwa F. Abdi (email: fatwa.abdi@helmholtz-berlin.de; ffabdi@cityu.edu.hk)

Supplementary Note 1 – Homogeneous Hydrogenation Reaction

We first investigated the hydrogenation of itaconic acid with a water-soluble Rh/TPPTS catalyst complex in a semi-batch reactor (see methods section in the main text), with deionized water as the solvent. Itaconic acid contains an external pro-chiral double bond that can be hydrogenated at ambient conditions. During the reaction, one hydrogen molecule is added to the double bond of IA, and the progress of the hydrogenation reaction can be monitored by the measured consumption of hydrogen. Figure S1a shows the volume of hydrogen consumed as a function of time with various concentrations of Rh/TPPTS catalyst (c_{cat}). The appearance of plateaus indicates that the hydrogenation reaction is finished (i.e., full conversion). The associated percentage of IA conversion (X_{IA}), calculated using equation 1 in the main text, is also shown, from which the initial reaction rate, r_0, can be calculated. Here, we define r_0 as the reaction rate when 10% of IA has been hydrogenated (i.e., X_{IA} = 10%, see equation 2 in the main text) as shown in Figure S1b. Increasing the catalyst concentration results in a monotonous increase of r_0 simply because of the higher number of active centers. Although higher catalyst concentrations accelerate the reaction, it should be noted that with increasing reaction rate, the reaction performed in the semi-batch reactor at 1550 rpm would become gas/liquid film diffusion-limited. Figure S1c shows that r_0 increases with increasing IA concentration until c_{IA} = ~0.1 M. When the IA concentration is increased further, r_0 stays constant. We attribute this to the complete saturation of the active centers for IA hydrogenation. For a specific catalyst concentration of c_{cat}, a certain number of active centers is available for IA hydrogenation. Upon full occupancy of these centers, no more IA can coordinate to the catalyst complex, and r_0 cannot increase any more even when the IA concentration is increased. A maximum hydrogenation rate of 2.4 mM/min is obtained when c_{cat} is 0.9 mM and c_{IA} is above 0.07 M.

Because ion conduction and the suppression of local pH change are essential for the targeted coupled (photo)electrochemical hydrogenation, we also performed the hydrogenation reaction in an aqueous potassium phosphate buffer solution (KP_i, pH = 7), which is the electrolyte to be used in the coupled (photo)electrochemical reaction. The hydrogenation performance in phosphate buffer solution was found to be comparable—even slightly faster—to that in pure water (Figure S1d). The catalyst complex is stable as evident from the absence of deactivation during the reaction.

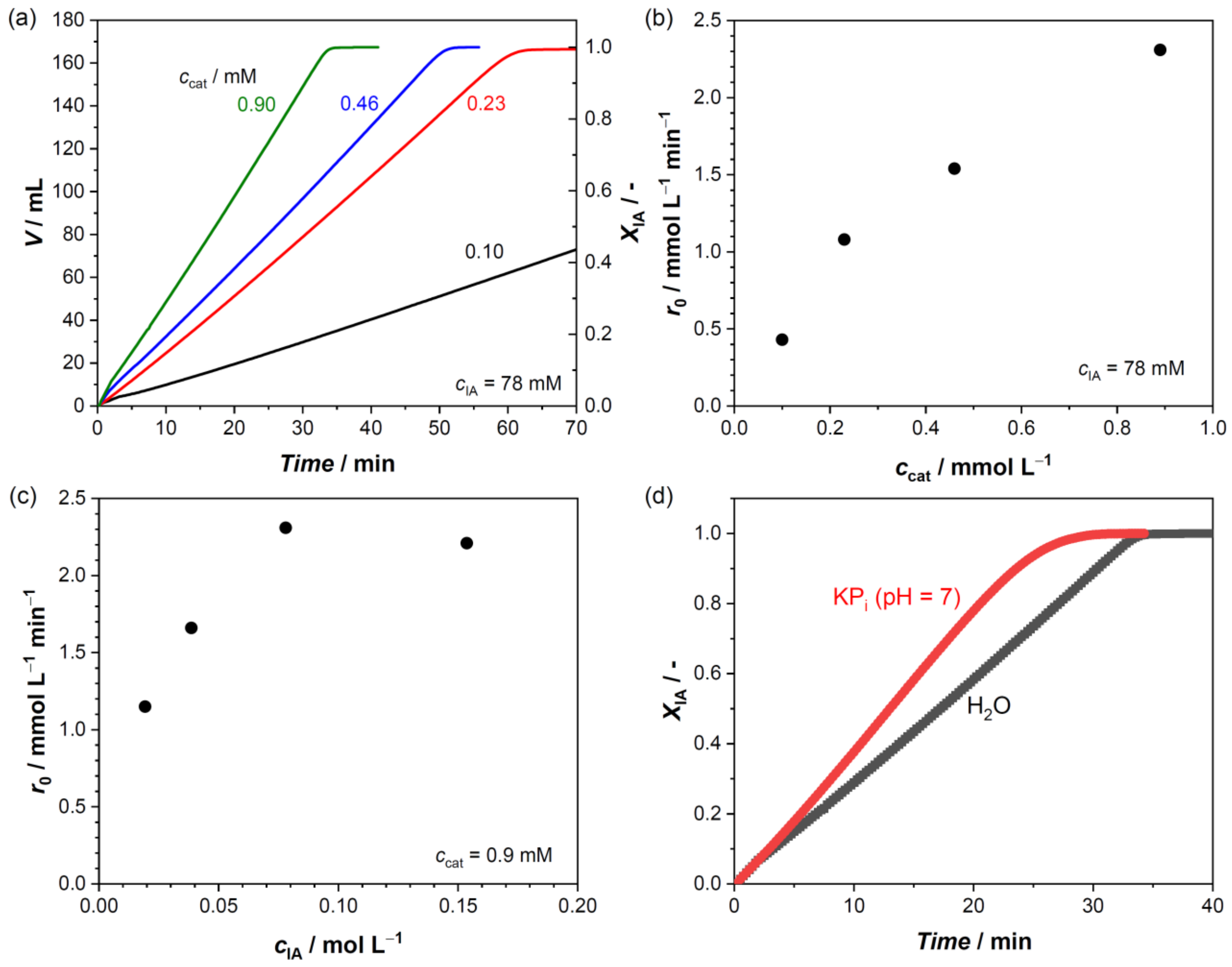

Figure S1. (a) Cumulative hydrogen consumption and the corresponding conversion of IA with various concentration of Rh/TPPTS catalyst, c_{cat}, (n_{Rh}:n_{TPPTS} = 1:7), and c_{IA} = 78 mM during the homogeneous hydrogenation in a semi-batch reactor. The initial reaction rates of the hydrogenation of itaconic acid are plotted with the variation of (b) Rh/TPPTS concentration (c_{IA} was kept constant at 78 mM), and (c) IA concentration (c_{cat} was kept constant at 0.9 mM). (d) The conversion of IA in pure H_2O and KP_i solutions (c_{cat} = 0.9 mM, and c_{IA} = 78 mM). In all experiments, the temperature (T = 25°C), total pressure (0.11 MPa), and stirrer speed (1550 rpm) were kept constant.

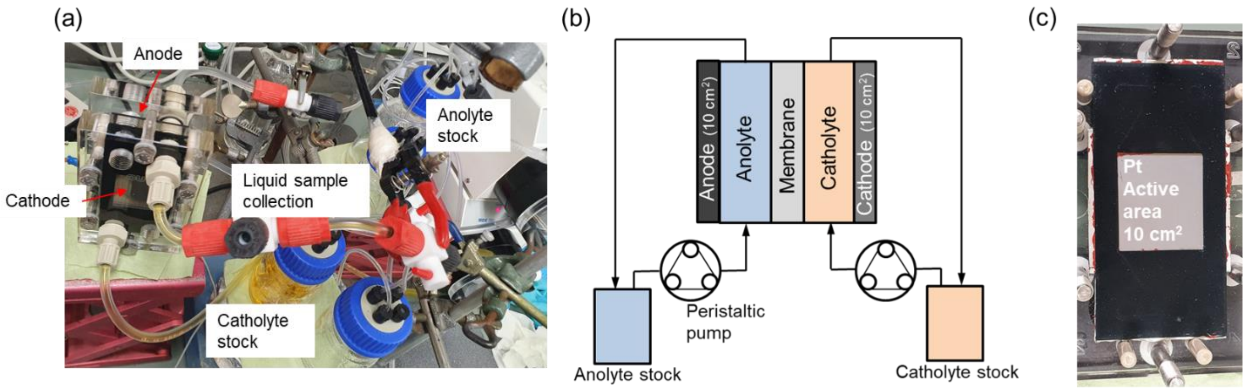

Figure S2. (a) Photograph and (b) simplified schematic of the flow cell used in our electrochemical experiments. (c) Geometry of the electrode indicating the 10 cm^2 active area.

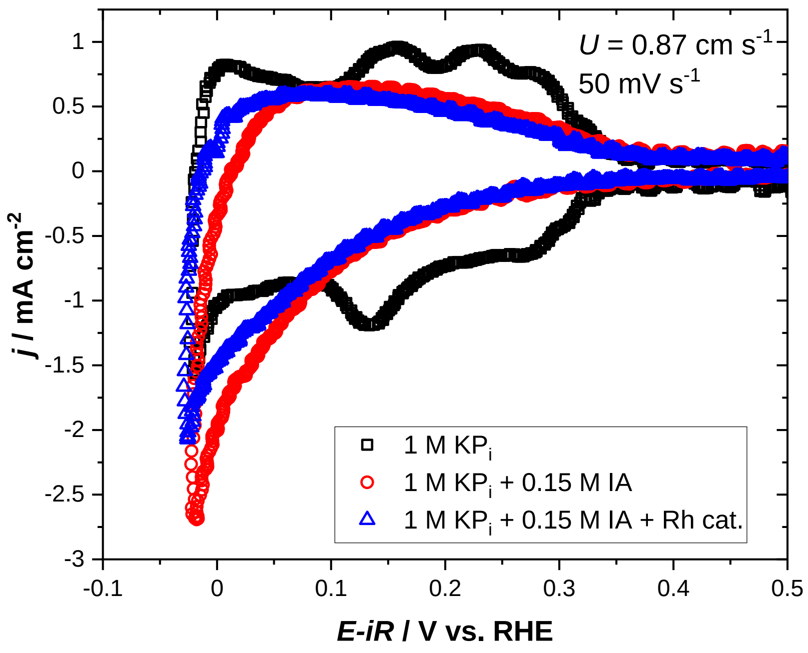

Figure S3. Cyclic voltammograms of a Pt electrode in 1 M KP_i with and without various additives: itaconic acid (IA) and Rh/TPPTS catalyst (Rh cat.). The region showing Pt-H underpotential deposition (UPD) peaks is shown. The measurements were performed with a scan rate of 50 mV/s and electrolyte inlet velocity of 0.87 cm/s.

Supplementary Note 2 – Underpotential Deposition Cyclic Voltammetry

The adsorption of organic molecules (i.e., IA and Rh/TPPTS) on Pt surface was analyzed from the Pt-H underpotential deposition (UPD) peaks in the cyclic voltammograms (CV). In KP_i, well-known Pt polycrystalline features are observed (black curve in Fig. S3). The sharp peaks located at 0.1 and 0.2 V vs. reversible hydrogen electrode (RHE) originate from the (110) and (100) facets, respectively,[1,2] and the broad background peak between 0 and 0.3 V vs. RHE can be attributed to the (111) facet.[2] In the presence of IA (red curve in Fig. S3), the broad peak remains, but the UPD peaks due to (110) and (100) disappear indicating preferential adsorption of IA on these facets.

This observation agrees with a previous report on the adsorption of various organic molecules on Pt surface.[3] No additional redox peak is observed within the potential range upon the addition of IA. Further introduction of Rh/TPPTS to the catholyte does not result in any changes (blue curve in Fig. S3).

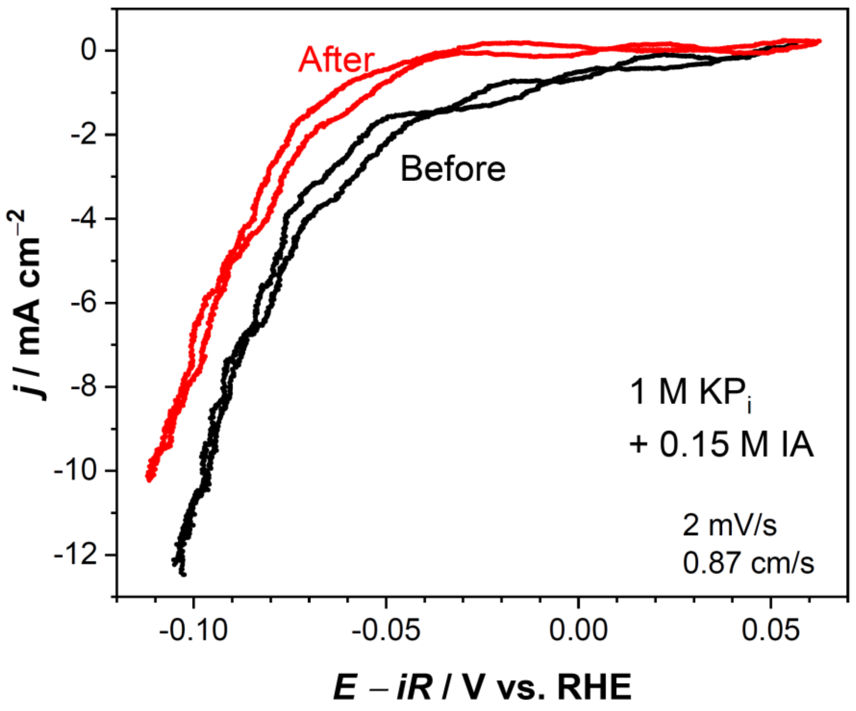

Figure S4. Cyclic voltammograms of Pt electrode in 1 M KP_i + 0.15 M IA before and after chronopotentiometry at −2 mA cm^{-2} for 90 min.

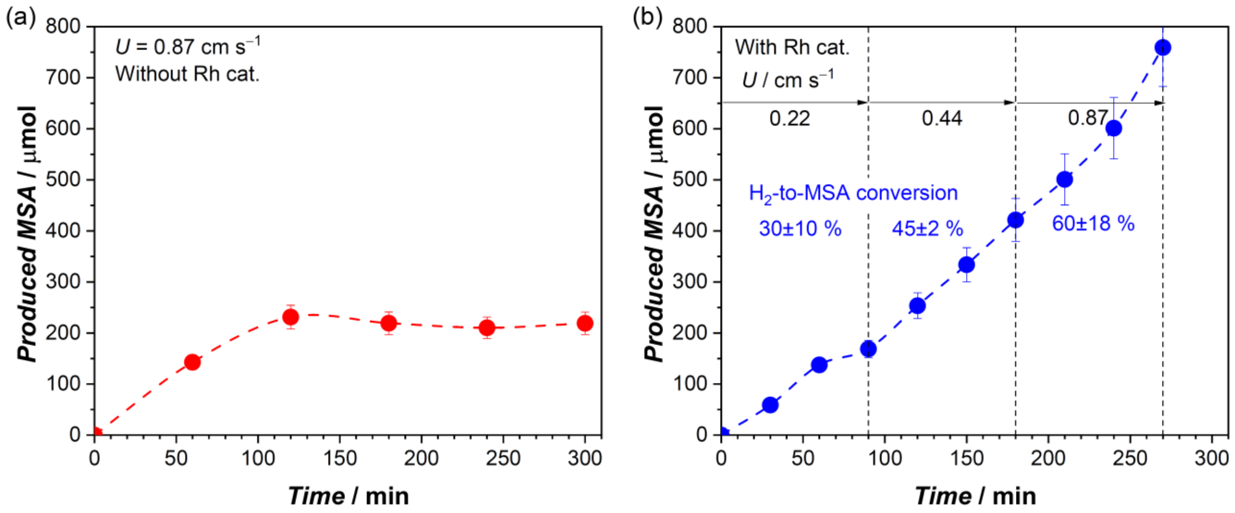

Figure S5. The amount of MSA produced (a) without and (b) with the Rh/TPPTS catalyst at -2 mA cm^{-2}. The velocity of the electrolyte is (a) 0.87 cm/s and (b) 0.22 - 0.87 cm/s. Error bars for produced MSA were estimated as 10% based on the data points in Fig. S5a at > 120 mins where the production of MSA has terminated. Error estimates of the H_2-to-MSA conversion were calculated based on the four data points for each electrolyte velocity.

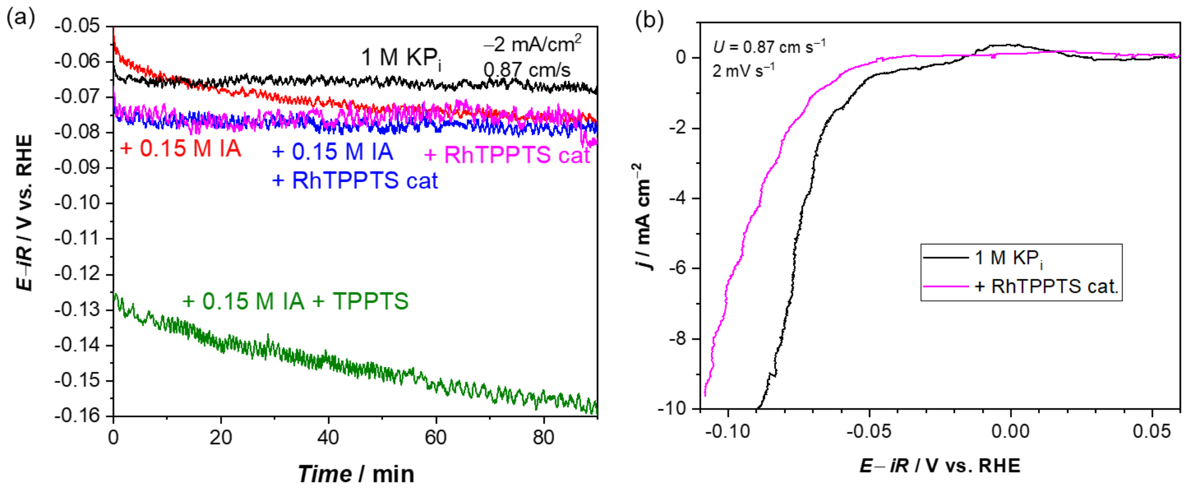

Figure S6. (a) Chronopotentiometry at −2 mA cm^{-2} with various additives in the electrolyte. (b) Linear sweep voltammograms of a Pt cathode in 1 M KPi, with and without Rh/TPPTS catalyst. The Pt electrode shows stable H_2 production in 1 M KP_i, as shown by the black curve. When IA is added to the electrolyte without any Rh/TPPTS catalyst (red and green curves), a gradual decrease of the measured potential is observed indicating that the heterogenous hydrogenation is deactivated during the course of the cathodic reactions. Only in the presence of Rh/TPPTS catalyst (blue and magenta curves) are the cathodic potentials stable suggesting the prevention of the heterogeneous hydrogenation reaction and the suppression of the deactivation of the Pt electrode. It is also noted that the HER overpotential without IA (i.e., in pure KP_i) increases in the presence of Rh/TPPTS catalyst (magenta curve vs. black curve), which implies the passivation of highly active HER sites on the Pt surface, such as the (110) and (100) facets, which are also adsorption sites of IA as discussed in Figure S3.

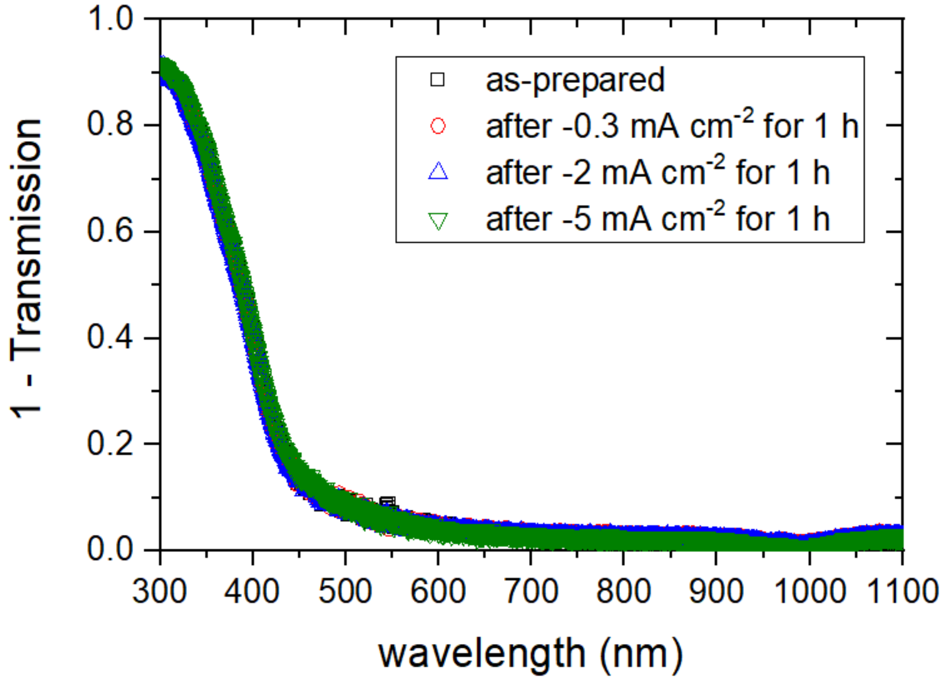

Figure S7. UV-vis absorptance (1 – Transmission) of the catholyte (1 M KP_i + 0.15 M IA + 0.9 mM Rh/TPPTS catalyst) at various stages: as-prepared (i.e., fresh) and after exposure to various current densities for 1 hour each. No change in absorptance indicates the stability of the Rh/TPPTS catalyst, as it was reported previously that Rh/TPPTS instability is accompanied by a color change.[4–6]

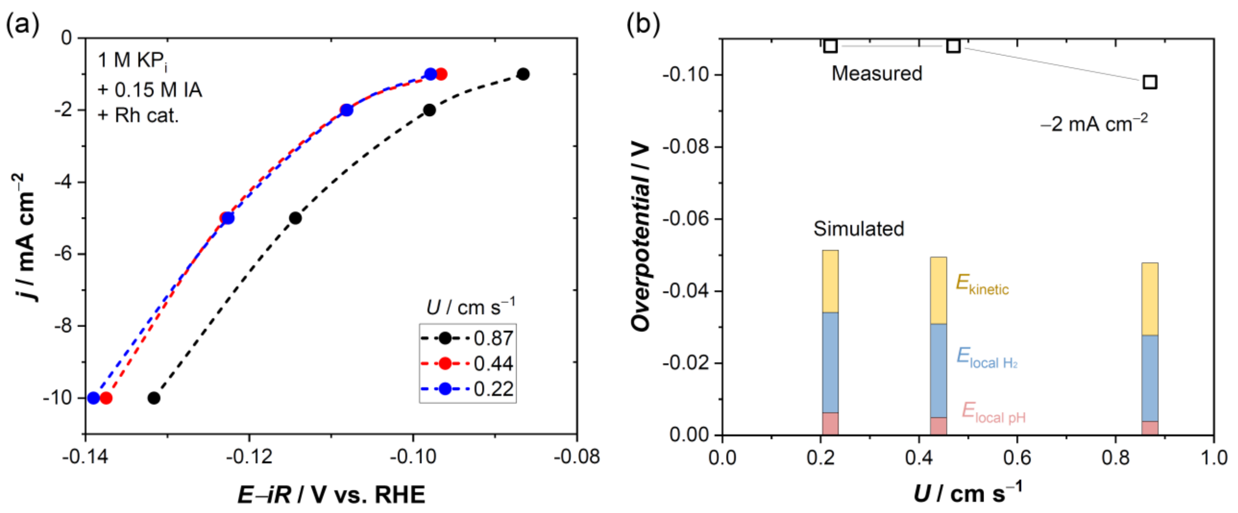

Figure S8. (a) Current-voltage response from the stabilized chronopotentiometry data in 1 M KPi containing IA and Rh catalysts at various electrolyte flow rates. (b) Comparison of the measured and numerically simulated overpotentials.

Supplementary Note 3 – Overpotentials vs. Flow Rate

The measured overpotentials change slightly (~10 mV) in the studied range of flow rates (Fig. S8a). Our numerical simulation results show that this minor decrease of overpotential originates mainly from the accumulated H_2 close to the Pt surface ($E_{local\ H_2}$, see Fig. S8b), which is consistent with previous reports.[1,7,8] The deviation between the measured and simulated overpotentials most likely originates from a decrease in the effective exchange current density due to the partial coverage of IA and/or TPPTS on the Pt surface, as discussed in Fig. S3 and Supplementary Note 2.

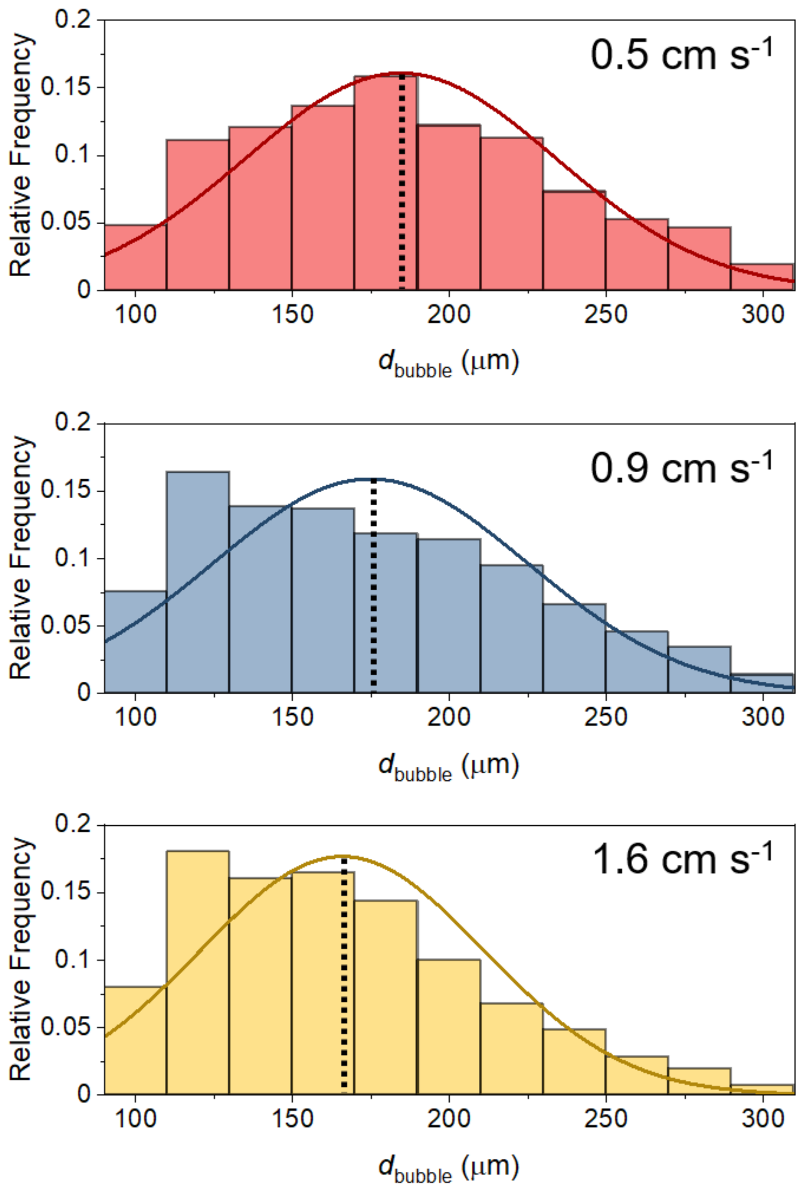

Figure S9. Relative frequency histogram of the H_2 bubble diameter (d_{bubble}) generated from the Pt cathode at various electrolyte velocities: 0.5 cm s^{-1} (top panel), 0.9 cm s^{-1} (middle panel), and 1.6 cm s^{-1} (bottom panel). The data were obtained from shadowgraphy experiments performed in 1 M KP_i electrolytes at −5 mA cm^{-2}. The normal distribution curves are also shown in each panel. The black horizontal dashed lines indicate the average bubble diameter value, which decreases from 184 to 166 μm as the electrolyte velocity increases from 0.5 to 1.6 cm s^{-1}.

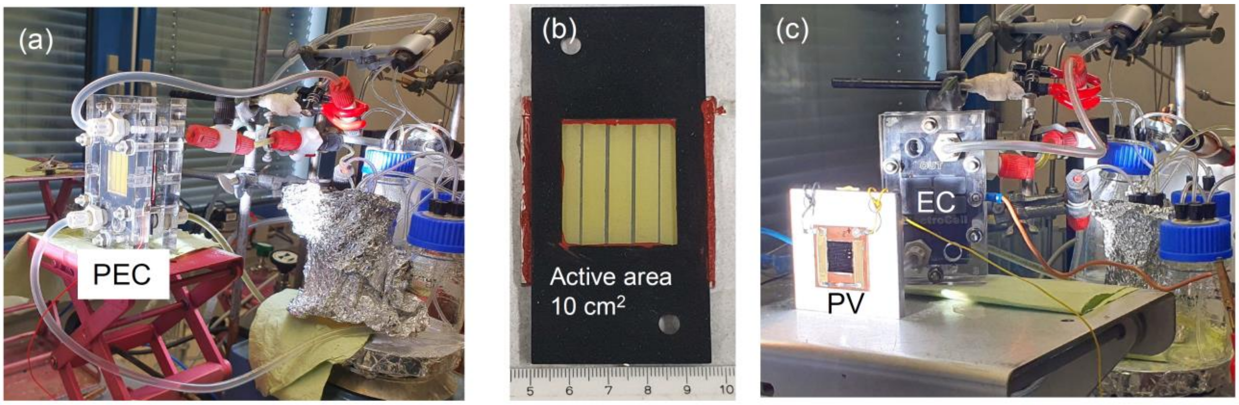

Figure S10. Photograph of solar-driven hydrogenation devices (a) with photoelectrochemical (PEC) cell and (c) with photovoltaic (PV) cell and electrochemical (EC) cell. (b) Photograph of W:$BiVO_4$ on an FTO substrate with Ni lines.

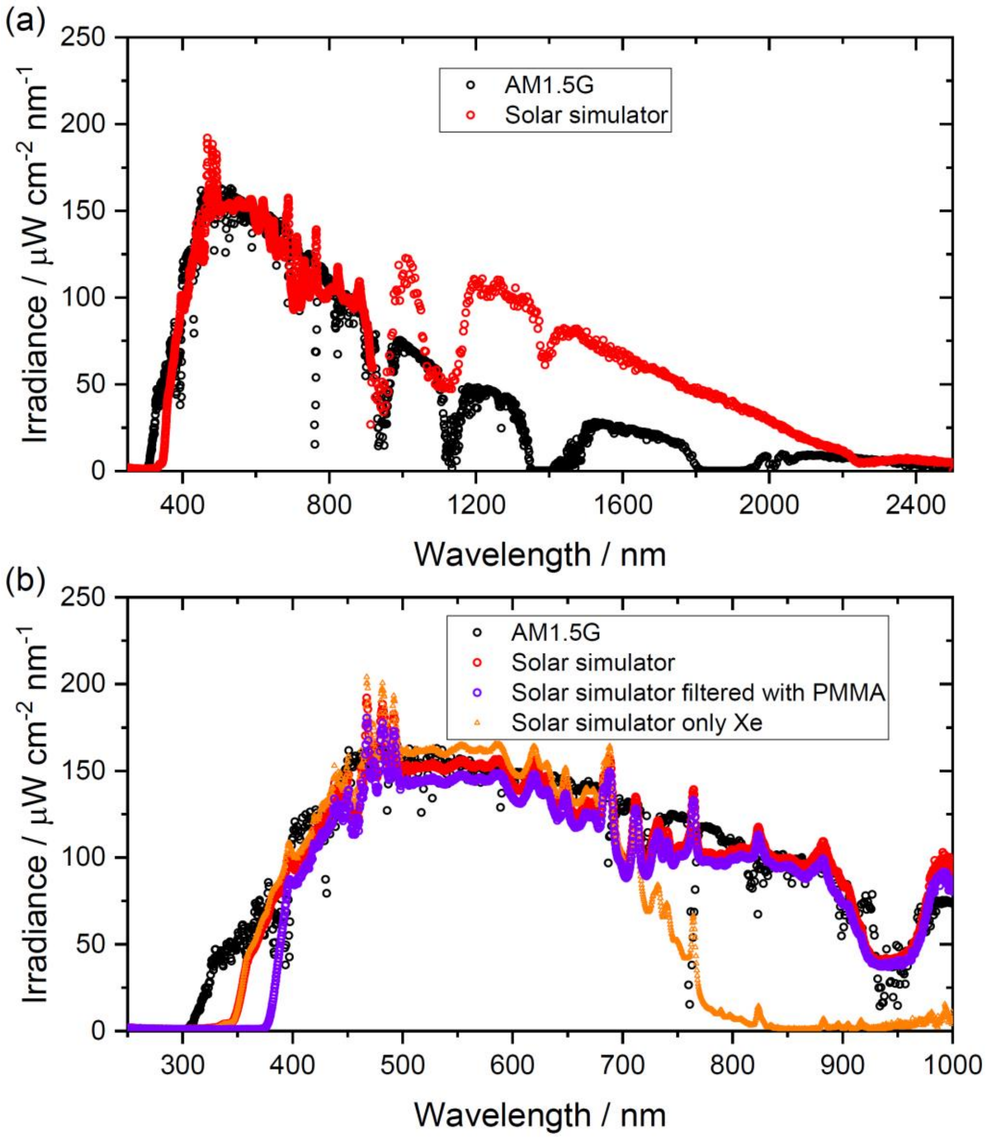

Figure S11. Solar spectrum used for demonstration. The full spectrum (from Xe and halogen lamps) is used for PV+EC demonstration (a) while only Xe lamp is used for the PEC demonstration (b). The irradiance in the wavelength range from 350 nm to 400 nm is reduced due to the PMMA frame during PEC demonstration. The spectra were measured using a calibrated spectrometer (USB2000+ and NIRQUEST+2.5, Ocean Insight)

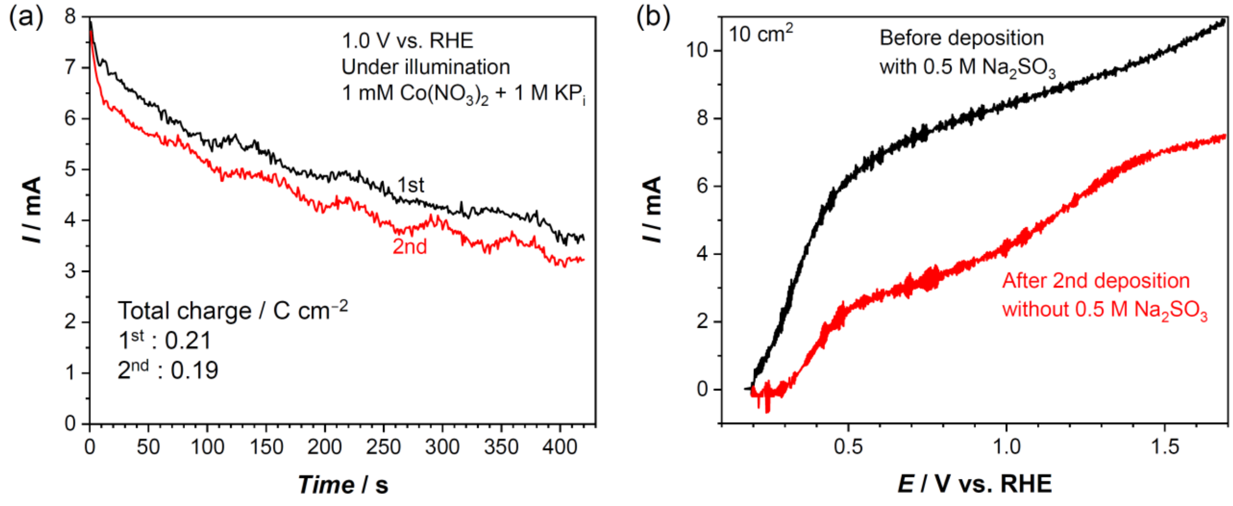

Figure S12. (a) Photocurrent during the photodeposition of CoP_i on our 10 cm^2 $BiVO_4$ photoanode. (b) Linear sweep voltammograms of CoP_i/W:$BiVO_4$ in 1 M KP_i with and without Na_2SO_3 sacrificial agent under light irradiation with the spectrum shown in Fig. S11b (Solar simulator filtered with PMMA). The catalytic efficiency of CoP_i-modified $BiVO_4$ was 60% at 1.2 V vs. RHE.

Table S1. H_2 collection efficiency and H_2-to-MSA conversion efficiency values obtained from a Pt electrode (in dark conditions) at the operating current levels relevant for the coupled PEC and PV-EC hydrogenation devices reported in this study. The electrolyte was 1 M KP_i with 0.15 M itaconic acid (IA) and 0.9 mM Rh/TPPTS catalyst. H_2 collection and H_2-to-MSA conversion efficiency values were evaluated using mass spectrometry and ^{1}H-NMR, respectively. Adding these two efficiency values yields the total Faradaic efficiency.

Current (mA)	H_2 collection efficiency (%)	H_2-to-MSA conversion efficiency (%)	Total Faradaic efficiency (%)
–3	34	70 ± 9	104 ± 9
–50	81	10 ± 6	91 ± 6

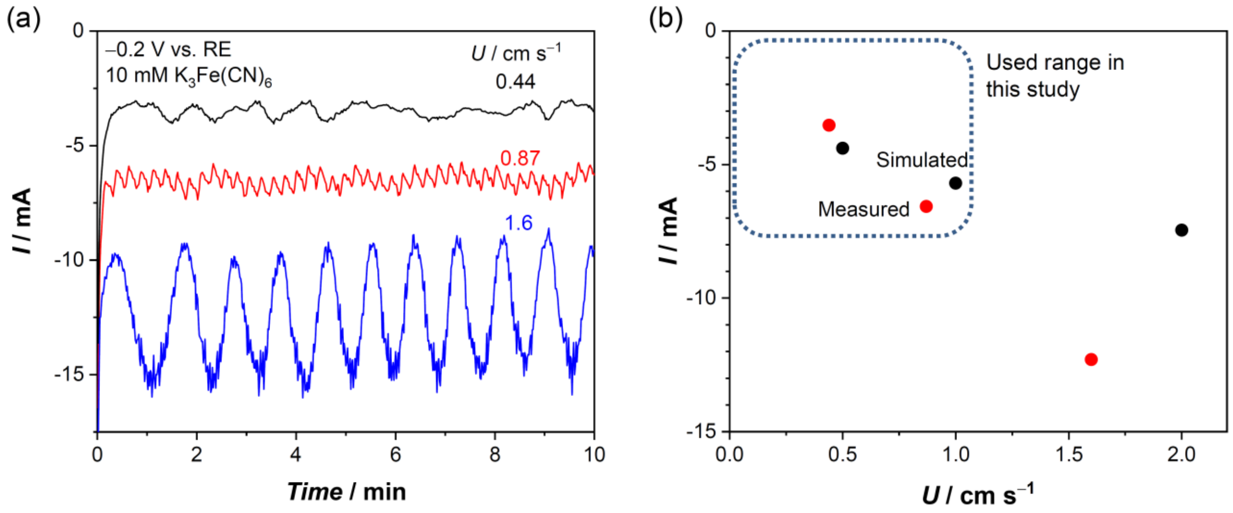

Figure S13. (a) Mass-transport limiting current of $Fe(CN)_6^{3-}$ in the electrochemical flow cell at various electrolyte velocities. The origin of the large fluctuation at an inlet velocity >0.87 cm s^{-1} is not clear at the moment, but it is expected to be specific to our experimental setup. (b) Comparison with the simulated limiting current. Considering the stability of flow, flow range is limited up to 0.87 cm s^{-1} in this study.

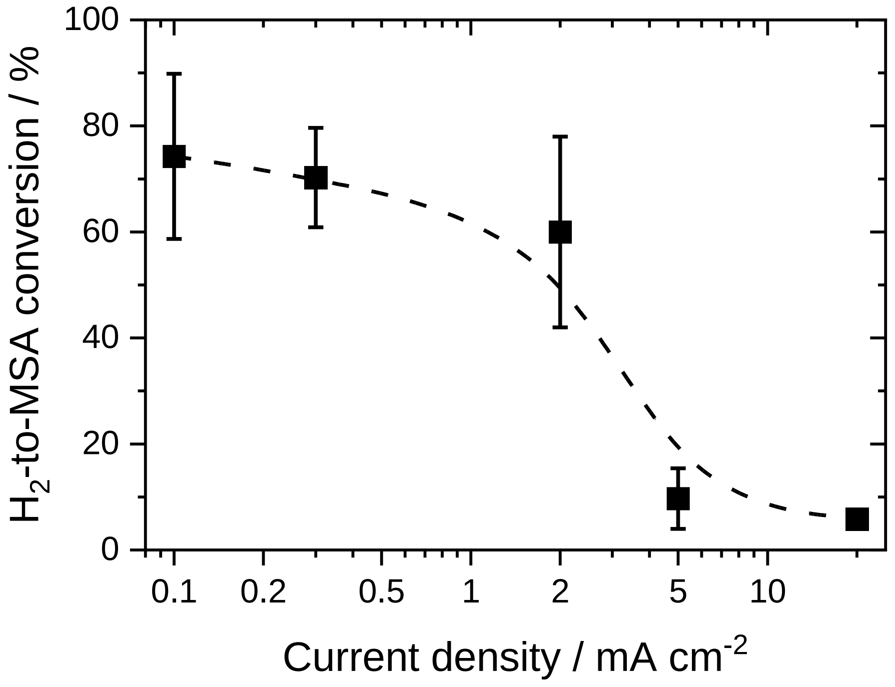

Figure S14. H_2-to-MSA conversion values obtained from a Pt electrode (in dark conditions) as a function of the current density. The electrolyte was 1 M KP_i with 0.15 M itaconic acid (IA) and 0.9 mM Rh/TPPTS catalyst. Error bars for each datapoints were calculated from at least three separate measurements.

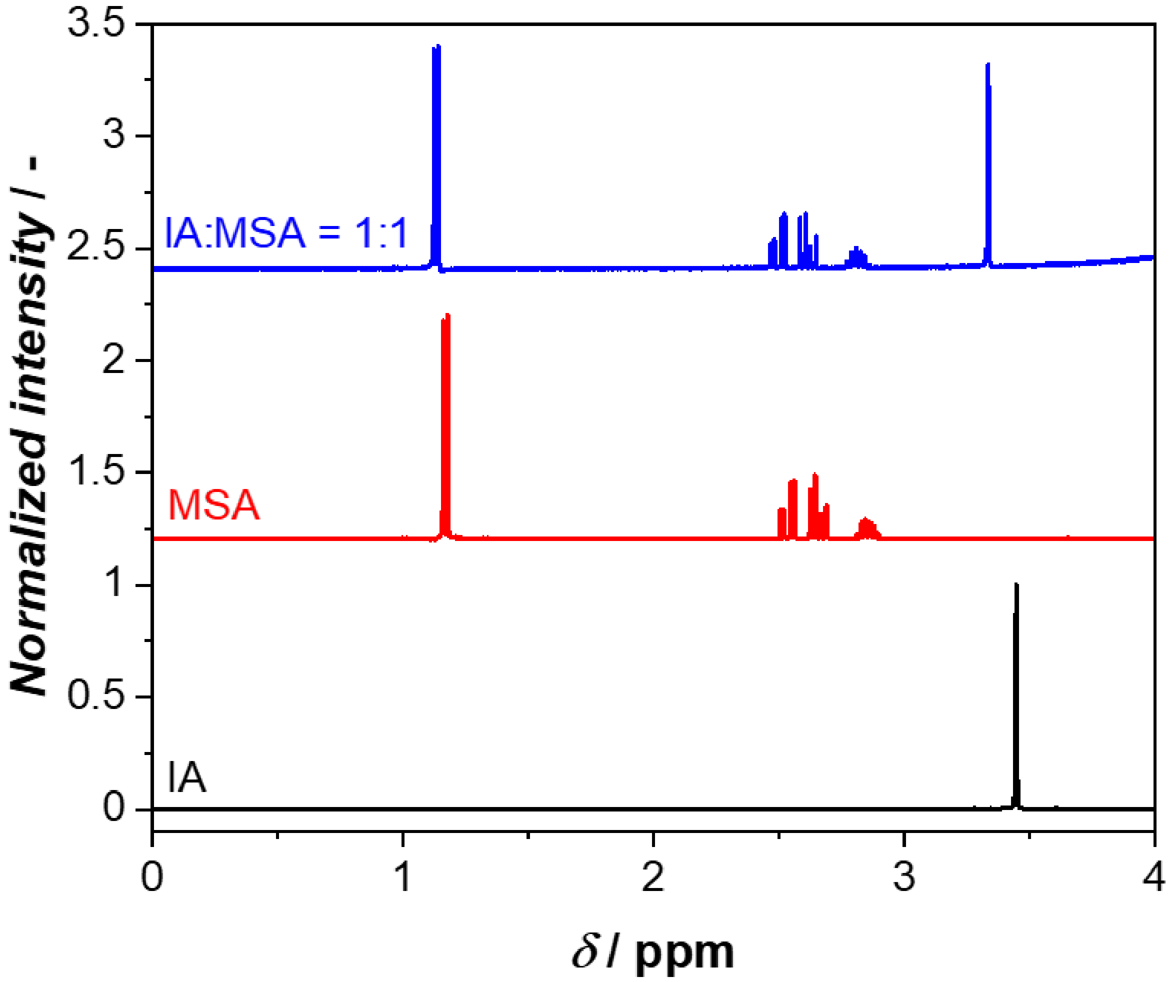

Figure S15. ^{1}H-NMR spectra of pure solutions (IA and MSA) and a mixture solution. The peaks around 1.1 ppm and 3.4 ppm are attributed to the doublet from $-CH_3$ in MSA and the singlet of $=CH_2$ in IA, respectively. Their peak areas were used to estimate the conversion from IA to MSA.

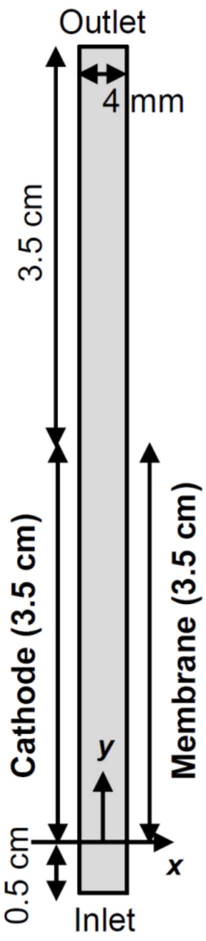

Figure S16. Geometry of the catholyte channel used in the numerical simulations.

Supplementary Note 4 – Numerical Model Description

A 2D geometry was defined based on the geometry of electrochemical flow cell as shown in Fig. S16. Mass-transport of dissolved species was simulated by solving Nernst-Planck equations with charge neutrality ($\Sigma Z_i c_i = 0$) at the steady state condition.

$$\boldsymbol{N}_i = -D_i \nabla c_i + \frac{-Z_i F}{RT} D_i c_i \nabla \phi_l + c_i \boldsymbol{v} \quad \text{(S1)}$$

$$\frac{\partial c_i}{\partial t} = -\nabla \cdot \boldsymbol{N}_i = 0 \quad \text{(S2)}$$

where $\boldsymbol{N}_i$, D_i, c_i, and Z_i represent the molar flux vector, the diffusion coefficient, the concentration, and the charge of species i, respectively. F, R, T, and ϕ_l are Faraday constant, gas constant, temperature, and the electrolyte potential, respectively. $\boldsymbol{v}$ is the velocity vector of the liquid phase, which is assumed to be fully developed laminar flow.

$$v_x = 0 \quad \text{(S3)}$$

$$v_y = 1.5U(1 - 2x/L_x)^2 \quad \text{(S4)}$$

where U and L_x are the average inlet velocity and the width of cathode channel, respectively. Local equilibrium of the buffer species was also assumed.

$$\frac{c_{\mathrm{H^+}} c_{\mathrm{HPO_4^{2-}}}}{c_{\mathrm{H_2PO_4^-}}} = K_{a2} \quad \text{(S5)}$$

K_{a2} is the second equilibrium constant of phosphate buffer. Minority buffer species (H_3PO_4 and PO_4^{3-}) are not considered. The homogeneous reaction rate, r, in the electrolyte determines the molar consumption of IA and H_2, and the molar production of MSA.

$$r = k_{\mathrm{Rh}} c_{\mathrm{Rh}} c_{\mathrm{H_2}} \quad \text{(S6)}$$

where k_{Rh} is the rate constant of homogeneous reaction. $k_{Rh}\,c_{Rh}$ is assumed to be constant and experimentally obtained from the homogeneous hydrogenation reaction (Fig. S1). At the cathode surface, the molar fluxes were determined by the local current density (j_s) and the stoichiometry coefficients (ν_i).

$$\boldsymbol{n} \cdot \boldsymbol{N}_i = \frac{-\nu_i j_s}{nF} \tag{S7}$$

where $\boldsymbol{n}$ is the normal vector. The stoichiometry coefficients for H^+ and H_2 are −2, 1, respectively, when the number of electrons involved (n) is 2.

$$\nu_{Ox} Ox + ne^- \rightleftharpoons \nu_{Red} Red \tag{S8}$$

The local electrode current density (j_s) was determined by concentration dependent Butler-Volmer equation.

$$j_s = j_{0,loc} \left\{ exp\left(\frac{\alpha_a F \eta}{RT}\right) - exp\left(\frac{-\alpha_c F \eta}{RT}\right) \right\} \tag{S9}$$

$j_{0,loc}$, α_a and α_c are local exchange current density, anodic and cathodic transfer coefficient, respectively. The overpotential (η) was determined by the following equation, which contains concentration overpotential due to the pH gradient.

$$\eta = \phi_s - \phi_l - 0 + \frac{RT}{2F} \ln\left\{ \left(\frac{c_{H^+,ref}}{c_{H^+}}\right)^2 \frac{c_{H_2}}{c_{H_2,ref}} \right\} \tag{S10}$$

The subscript *ref* corresponds to the reference condition (pH 7 and $c_{H2,\,ref}$ = 0.78 mM at 1 atm H_2). The local exchange current density is H_2-dependent and pH-independent.[8–11]

$$j_{0,loc} = j_{0,ref} \left(\frac{c_{H_2}}{c_{H_2,ref}}\right)^{\alpha_c} \tag{S11}$$

Table S2. Parameters used for the multiphysics simulations.

**Parameters for the electrolyte solutions**	**Value**	**Ref.**
H^+ concentration at the inlet, $c_{H^+,in}$	$1.0 \times 10^{-7.21}$ mol/L	
$H_2PO_4^-$ concentration at the inlet, $c_{H_2PO_4^-,in}$	0.5 mol/L	
HPO_4^{2-} concentration at the inlet, $c_{HPO_4^{2-},in}$	0.5 mol/L	
IA concentration at the inlet, $c_{IA,in}$	1.5 mol/L	
MSA concentration at the inlet, $c_{MSA,in}$	0 mol/L	
H_2 concentration at the inlet, $c_{H_2,in}$	0 mol/L	
Diffusion coefficient of H^+, D_{H^+}	9.3×10^{-9} m^2/s	[12]
Diffusion coefficient of K^+, D_{K^+}	1.96×10^{-9} m^2/s	[12]
Diffusion coefficient of $H_2PO_4^-$, $D_{H_2PO_4^-}$	0.85×10^{-9} m^2/s	[12]
Diffusion coefficient of HPO_4^{2-}, $D_{HPO_4^{2-}}$	0.69×10^{-9} m^2/s	[12]
Diffusion coefficient of H_2, D_{H_2}	5.0×10^{-9} m^2/s	[12]
Diffusion coefficient of MSA, D_{MSA}	9.4×10^{-9} m^2/s	[13]
Diffusion coefficient of IA, D_{IA}	Assumed to be = D_{MSA}	
$k_{Rh}\ c_{Rh}$	0.028 /s	
2nd buffer equilibrium constant, K_{a2}	$1.0 \times 10^{-7.21}$ mol/L	7
Dynamic viscosity, μ	9.0×10^{-4} Pa s	[12]
Temperature, T	298 K	

**Parameter for electrochemistry**	**Value**	**Ref.**
Exchange current density for HER, $j_{0,ref}$	1 mA/cm^2	8,12
HER anodic transfer coefficient, $\alpha_{a,HER}$	0.5	
HER cathodic transfer coefficient, $\alpha_{c,HER}$	0.5	

Supplementary Note 5 – Control Photochemical Measurement

Since $BiVO_4$ transmits photons with wavelength > 500 nm, one may argue that the hydrogenation of IA to MSA may be caused by a photochemical reaction of the Rh catalysts and not our desired coupled reaction. In order to evaluate this possibility, a control measurement under solar irradiation was performed without any electrical connections between the $BiVO_4$ photoanode and the cathode (Figure S17), i.e., photochemistry of the Rh homogeneous catalysts using $BiVO_4$ as a filter. Under this configuration, MSA could not be detected even after 2 h of solar irradiation, which confirms that the obtained MSA in Fig. 4c is a result of a coupled reaction between the photoelectrochemical H_2 generation and in-situ hydrogenation of IA.

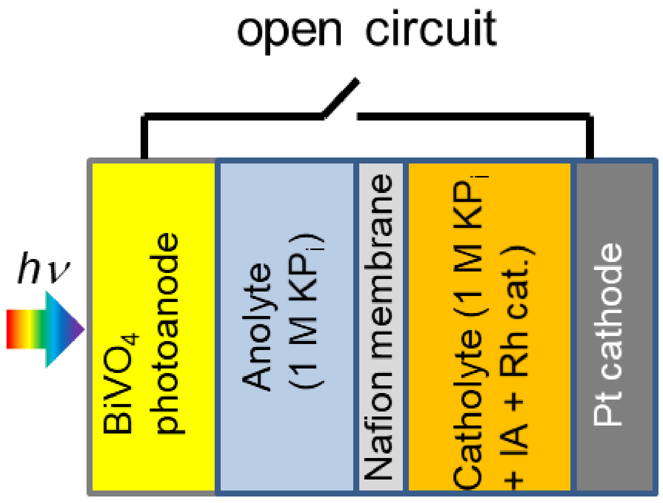

Figure S17. Schematics representation of the control photochemical experiment using $BiVO_4$ as a filter.

Supplementary Note 6 –Techno-economic Assessment

The techno-economic assessment in this study was performed following the principles and framework described in “Techno-Economic Assessment (TEA) & Life Cycle Assessment (LCA) Guidelines for CO_2 Utilization (version 1.1)” established by the Global CO_2 Initiative. [17] The device considered is based on a $BiVO_4$-based photoanode as the top absorber and a silicon heterojunction (SHJ) solar cell as the bottom absorber, as reported in the literature[18] and also considered in the experimental demonstration of this study. The scope of the assessment is cradle-to-gate (see Fig. S18), which covers the materials and fabrication processes of the device-scale components as well as the operation feedstocks. Balance-of-system (BOS) components (e.g., piping, separation, wiring), maintenance, and end-of-life treatment were also considered. The average solar insolation of Germany (3.4 kWh/day/m^2) was considered in our study.

The different materials and parameters considered for the various components of our coupled photoelectrochemical device is shown in Table S2, which were used to perform the life cycle inventory and calculate the primary cost of our device. Both the cost for upstream (raw) materials production as well as fabrication process were used. Data on several common materials (e.g., metals, plastics, acids) and SHJ components that have already been previously investigated by techno-economic assessments are available in the literatures; these data were thus directly used in our analysis. For certain materials and processes not available in the literatures, we built their individual life cycle inventories using estimates and processes from laboratory data and parameters. The cost breakdown for each component is also shown in Table S2, and the total primary cost of our photoelectrochemical device is 367 € m^{-2}.

The annual net profit, as shown in Table 1 in the manuscript, were calculated by subtracting the normalized annual cost (C) from the annual revenue (R).

$$\text{Annual net profit} = R - C \tag{S12}$$

$$R = \frac{\sum_{i=1}^{n} \frac{\Phi_{H_2,i} \times \text{price}_{H_2}}{(1+r)^i} + \sum_{i=1}^{n} \frac{\Phi_{MSA,i} \times \text{price}_{MSA}}{(1+r)^i}}{n} \tag{S13}$$

$$C = \frac{C_{capex} + \sum_{i=1}^{n} \frac{C_{O\&M,i}}{(1+r)^i} + \frac{C_{decom}}{(1+r)^n}}{n} \tag{S14}$$

$\Phi_{H_2,i}$ and $\Phi_{MSA,i}$ are the annual amount of H_2 and MSA generated by the device in year i, calculated based on the η_{STH}, H_2-to-MSA conversion and average solar insolation. The price of H_2 was taken as 9.16 € kg^{-1}, based on the recent price of green hydrogen in Germany.[19] The price of MSA was taken as 13.5 € kg^{-1}, based on the market price of IA[20] and the energetic ratio between IA to MSA.[21] C_{capex} is the capital cost (see Tables S3 and S4), $C_{O\&M,i}$ is the annual operation and maintenance (O&M) cost in year i (see Table S4), n is the device lifetime, r is the annual interest rate (2.16%), and C_{decom} is the decommissioning cost (see Table S4). As an example, the costs of the different system components are listed in Table S5 for the case of n = 10 years.

The levelized cost of hydrogen (LCOH), as shown in Table 1, were calculated using the substitution method as described in the "Techno-Economic Assessment (TEA) & Life Cycle Assessment (LCA) Guidelines for CO_2 Utilization (version 1.1)" established by the Global CO_2 Initiative. [17] In short, we assumed that the revenue obtained from the produced MSA is used to compensate the cost of the whole system. At such, a negative LCOH value may be obtained (which is the case as shown in Table 1); this indicates that the revenue obtained from the produced MSA exceeds the total cost of the system.

$$LCOH = \frac{C_{\text{capex}} + \sum_{i=1}^{n} \frac{C_{\text{O\&M},i}}{(1+r)^{i}} + \frac{C_{\text{decom}}}{(1+r)^{n}} - \sum_{i=1}^{n} \frac{\Phi_{\text{MSA},i} \times \text{price}_{\text{MSA}}}{(1+r)^{i}}}{\sum_{i=1}^{n} \frac{\Phi_{\text{H}_2,i}}{(1+r)^{i}}} \quad \text{(S15)}$$

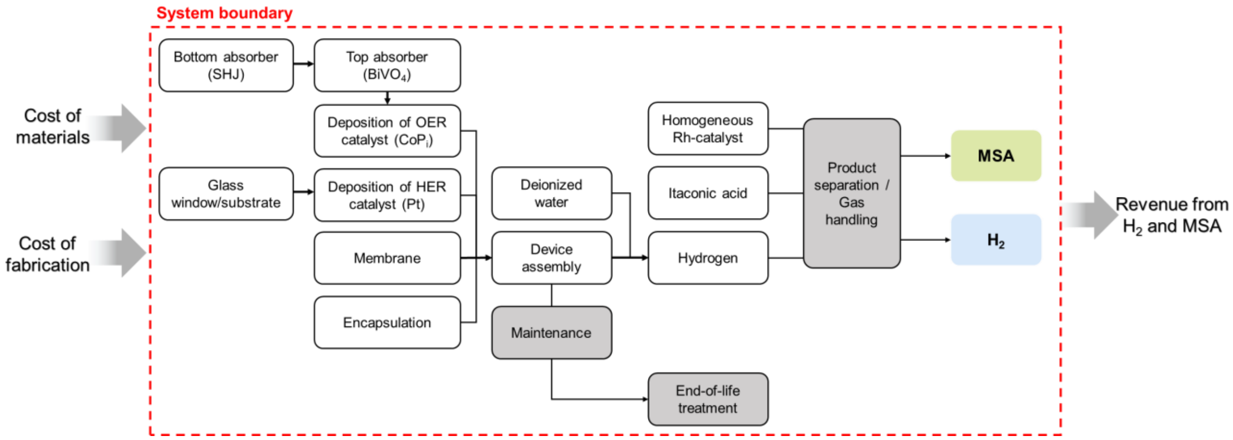

Figure S18. Simplified process flow diagram for the techno-economic analysis of our coupled photoelectrochemical hydrogenation device. The dashed red line indicates the system boundary or scope of this study (cradle-to-gate), from raw material extraction to product separation and the end-of-life treatment of the device.

Table S3. Materials and parameters for the coupled photoelectrochemical hydrogenation device considered in the TEA study. The breakdown of the cost that in total forms the primary cost of the device is also shown.

Component	Materials & Parameters		Data source	Cost (€ m^{-2})
**Photoelectrodes**				
Bottom absorber	SHJ cell	180 μm	Literature[22]	66
Top absorber	$BiVO_4$ (spray pyrolysis)	200 nm	Laboratory data, literature [18]	44
HER catalyst	Pt (electrodeposition)	20 nm	Literature[23,24]	4
OER catalyst	Co-P_i (electrodeposition)	30 nm	Literature [23]	12
**Membrane**				
Membrane material	Nafion	50 μm	Literature [23]	203
Intervening layer	PEDOT-PSS	20 μm	Literature [23]	0
**Hydrogenation catalyst**	Rh/TPPTS	2.5 mmol m^{-2}	Laboratory data, literature[25]	5
**Encapsulation**				
Cover	Solar grade glass	5 mm	Literature [23,25]	4
Chamber	Polyvinyl chloride (PVC)	5 mm	Literature [23,25]	11
**Ancillary processes**				
Environmental control			Literature [23,26]	15
Water pumping and cleaning			Literature [23]	3
Miscellaneous chemicals			Literature [23]	0.3
**Total**				**367**

Table S4. Further assumptions considered for the coupled photoelectrochemical hydrogenation device.

System components	Assumptions	Ref.
Balance-of-system		
Gas handling	9.6% of H_2 production cost	[27]
Separation unit	16% of coupled process cost	[28]
O&M		
Annual replacement	10% of PEC device	[29]
IA feedstock	1.5 €/kg	[20]
Water usage	0.2 kg/day/m^2, water tariff at 1.8 €/m^3	[30]
Water delivering	0.01 kWh/m^3·km, pump powered by country mix electricity	[31]
Gas handling	2 kWh/kg produced H_2	[27]
MSA separation	0.36 €/kg produced MSA	[28]
End-of-life treatment		
Decommissioning of plant	10% of capital cost	[27]

Table S5. Example values for cost of inputs parameters (Tables S3 and S4) considering a 100 m^2 coupled PEC system with a lifetime of 10 years.

System components	Cost (€ m^{-2})
Primary	
PEC device	367
Balance-of-system	
Gas handling	7
Separation unit	60
O&M	
Annual replacement	367
IA feedstock	2,677
Water usage	2
Water delivering	0.01
Gas handling	16
MSA separation	652
End-of-life treatment	
Decommissioning of plant	43

Supplementary References

1. Sheng, W. *et al.* Correlating hydrogen oxidation and evolution activity on platinum at different pH with measured hydrogen binding energy. *Nat Commun* **6**, 5848 (2015).

2. Marković, N. M., Grgur, B. N. & Ross, P. N. Temperature-dependent hydrogen electrochemistry on platinum low-index single-crystal surfaces in acid solutions. *Journal of Physical Chemistry B* **101**, 5405–5413 (1997).

3. Singh, N. *et al.* Quantifying Adsorption of Organic Molecules on Platinum in Aqueous Phase by Hydrogen Site Blocking and in Situ X-ray Absorption Spectroscopy. *ACS Catal* **9**, 6869–6881 (2019).

4. Zhang, N., Yang, L. & Wnag, F. Selective Hydrogenation of Isophorone by Rh-TPPTS Catalysts. *Journal of Organic Chemistry Research* **04**, 13–22 (2016).

5. Schmidt, T. *et al.* Unusual Deactivation in the Asymmetric Hydrogenation of Itaconic Acid. *Adv Synth Catal* **351**, 750–754 (2009).

6. Schmidt, M. *et al.* Hydrogenation of Itaconic Acid in Micellar Solutions: Catalyst Recycling with Cloud Point Extraction? *Ind Eng Chem Res* **58**, 2445–2453 (2019).

7. Obata, K., Stegenburga, L. & Takanabe, K. Maximizing Hydrogen Evolution Performance on Pt in Buffered Solutions: Mass Transfer Constrains of H 2 and Buffer Ions. *The Journal of Physical Chemistry C* **123**, 21554–21563 (2019).

8. Zheng, J., Yan, Y. & Xu, B. Correcting the Hydrogen Diffusion Limitation in Rotating Disk Electrode Measurements of Hydrogen Evolution Reaction Kinetics. *J Electrochem Soc* **162**, F1470–F1481 (2015).

9. Durst, J., Simon, C., Hasché, F. & Gasteiger, H. A. Hydrogen Oxidation and Evolution Reaction Kinetics on Carbon Supported Pt, Ir, Rh, and Pd Electrocatalysts in Acidic Media. *J Electrochem Soc* **162**, F190–F203 (2015).

10. Rheinländer, P. J., Herranz, J., Durst, J. & Gasteiger, H. A. Kinetics of the Hydrogen Oxidation/Evolution Reaction on Polycrystalline Platinum in Alkaline Electrolyte Reaction Order with Respect to Hydrogen Pressure. *J Electrochem Soc* **161**, F1448–F1457 (2014).

11. Bagotzky, S. & Osetrova, N. v. Investigation of Hydrogen Ionization on Platinum. *Electroanalytical Chemistry* **43**, 233–249 (1973).

12. Lide, D. R. *Handbook of Chemistry and Physics*. (CRC Press, 2003).

13. Øyaas, J., Storrø, I., Svendsen, H. & Levine, D. W. The effective diffusion coefficient and the distribution constant for small molecules in calcium-alginate gel beads. *Biotechnol Bioeng* **47**, 492–500 (1995).

14. Green, A. A. The Preparation of Acetate and Phosphate Buffer Solutions of Known Ph and Ionic Strength. *J Am Chem Soc* **55**, 2331–2336 (1933).

15. Haussener, S. *et al.* Modeling, simulation, and design criteria for photoelectrochemical water-splitting systems. *Energy Environ Sci* **5**, 9922–9935 (2012).

16. Trasatti, S. Work function, electronegativity, and electrochemical behaviour of metals. *J Electroanal Chem Interfacial Electrochem* **39**, 163–184 (1972).

17. Zimmerman, A., Müller, L. & Wang, Y. *Deep Blue Home Login Home / Research Collections / Global CO2 Initiative / View Item Techno-Economic Assessment & Life Cycle Assessment Guidelines for CO2 Utilization (Version 1.1)*. http://hdl.handle.net/2027.42/162573 (2020) doi:10.3998/2027.42/162573.

18. Ahmet, I. Y. *et al.* Demonstration of a 50 cm2 BiVO4 tandem photoelectrochemical-photovoltaic water splitting device. *Sustain Energy Fuels* **3**, 2366–2379 (2019).

19. Conventional hydrogen yields slightly. *Hydex weekly report* https://www.energate-messenger.com/news/222655/conventional-hydrogen-yields-slightly (2022).

20. De Carvalho, J. C., MagalhÃes Jr., A. I. & Soccol, C. R. Biobased itaconic acid market and research trends - is it really a promising chemical? *Chim Oggi* **36**, 56 (2018).

21. Zhang, X., Schwarze, M., Schomäcker, R., van de Krol, R. & Abdi, F. F. Life cycle net energy assessment of sustainable H2 production and hydrogenation of chemicals in a coupled photoelectrochemical device. *Nat Commun* **14**, 991 (2023).

22. Louwen, A., van Sark, W. G. J. H. M., Schropp, R. E. I., Turkenburg, W. C. & Faaij, A. P. C. Life-cycle greenhouse gas emissions and energy payback time of current and prospective silicon heterojunction solar cell designs. *Progress in Photovoltaics: Research and Applications* **23**, 1406–1428 (2015).

23. Zhai, P. *et al.* Net primary energy balance of a solar-driven photoelectrochemical water-splitting device. *Energy Environ Sci* **6**, 2380 (2013).

24. Platinum 1968-2022 historical data . https://tradingeconomics.com/commodity/platinum.

25. Sigma-Aldrich price lists. https://www.sigmaaldrich.com/DE/en/product/aldrich.

26. Zhai, P. & Williams, E. D. Dynamic Hybrid Life Cycle Assessment of Energy and Carbon of Multicrystalline Silicon Photovoltaic Systems. *Environ Sci Technol* **44**, 7950–7955 (2010).

27. James, B. D., Baum, G. N., Perez, J. & Baum, K. N. *Technoeconomic Analysis of Photoelectrochemical (PEC) Hydrogen Production*. http://www.osti.gov/servlets/purl/1218403/ (2009) doi:10.2172/1218403.

28. Efe, Ç., van der Wielen, L. A. M. & Straathof, A. J. J. Techno-economic analysis of succinic acid production using adsorption from fermentation medium. *Biomass Bioenergy* **56**, 479–492 (2013).

29. Sathre, R. *et al.* Life-cycle net energy assessment of large-scale hydrogen production via photoelectrochemical water splitting. *Energy Environ. Sci.* **7**, 3264–3278 (2014).

30. Charge for drinking water supplied to tariff areas by tariff type. https://www.destatis.de/EN/Themes/Society-Environment/Environment/Water-Management/Tables/tw-07-charges-for-drinking-water-tariff-areas-2017-2019.html.

31. Plappally, A. K. & Lienhard V, J. H. Energy requirements for water production, treatment, end use, reclamation, and disposal. *Renewable and Sustainable Energy Reviews* **16**, 4818–4848 (2012).
